# The living medicine inside us: *in vitro* therapeutic prospects of human gut bacteria

**DOI:** 10.1080/29933935.2025.2480093

**Published:** 2025-04-03

**Authors:** K. M. Salim Andalib, Fabliha Bashashat Rodosy, Ahsan Habib

**Affiliations:** a Biotechnology and Genetic Engineering Discipline, Life Science School, Khulna University, Khulna, Bangladesh; b Department of Microbiology, Bhashasoinik Gaziul Haque Institute of Bioscience, Bogura, Bangladesh

**Keywords:** Gut bacteria, bacteria-derived metabolites, therapeutic prospects, biotherapeutics, gut health, probiotics

## Abstract

Gut microbial metabolism is intimately coupled to host health and disease. Recent knowledge on potential health benefits of gut microbiome lays the groundwork for development of novel therapeutic strategies. But how microbiota-derived metabolites impact on host-microbiome crosstalk remains untapped from therapeutic perspectives. In this study, six gut bacteria sourced from a fecal pool of forty healthy donors were cultured in three distinct growth media. Subsequently, the bacteria were identified through 16S rRNA gene sequencing and subjected to metabolite extraction to evaluate their anti-microbial, anti-oxidant and anti-thrombotic potential. Findings reveal strong anti-oxidant activities in the metabolic-extracts from all the isolates. Metabolites derived from *Lactobacillus rhamnosus*, *Priestia flexa* and *Bacillus subtiilis* inhibited the growth of clinically pathogenic strains *Escherichia coli* ATCC-8739, *Salmonella typhi* ATCC-1408 and *Staphylococcus aureus* ATCC-6538. *Escherichia fergusonii* originated metabolites demonstrated the highest efficacy in lysing blood clots compared to streptokinase. Additionally, extracts from all the isolates exhibited significant ability to delay coagulation time, competing with standard warfarin. Thus, the findings of our early-stage study provide novel insights into metabolomic functions of gut microbiota. This study underscores the significance of exploring these active metabolites for prospective therapeutic and clinical exploration at the intersection of drug discovery and live bio-therapeutics.

## Background

It is commonly stated that there are more bacteria in our gut than cells in our body.^1^ This large number of dynamic and complex bacterial ecosystems, in conjunction with other microorganisms are collectively referred as the “gut microbiome”.^2^ These diverse microbial communities colonize the human gastrointestinal tract (GIT) from our infancy and develop an intrinsic relationship with host biology.^3^ From birth, these bacteria play a crucial role in digestion, metabolism, and immunity.^4^ As we age, the composition and function of gut microbiota evolve and influence various aspects of host physiology.^5^ Since many studies have confirmed the role of gut bacteria beyond the digestive tract, extending to distant organs, extensive efforts have been made to comprehend the gut microbiota – host cross-talk over the last two decades.^5–7^ Advanced technologies like next generation sequencing or metagenomics have linked gut microbiota dysbiosis to complex diseases ranging from gastrointestinal complexities like inflammatory bowel disease or celiac diseases, to extra-intestinal disorders including arthritis, obesity, diabetes, allergy, autoimmune diseases, cardiovascular diseases, cancers, neurodegenerative and brain disorders.^7–10^ Findings have also revealed abundance or decrease of specific gut bacterial genus to be correlated with homeostasis or disease condition, reflecting their potential as biomarkers.^11^ For instance, the heightened presence of beneficial bacteria such *as Lactobacillus spp., Bacillus spp., Ruminicoccus spp., Akkermansia spp., Roseburia spp., Bifidobacterium spp., and Faecalibacterium spp*. in human gut is appreciated for their promising health benefits.^11–14^ Conversely, a reduced count of these bacterial populations has been reported in various disease conditions.^11,14^

These meticulous findings consequently led to development of some novel gut-microbiota-directed biotherapeutic strategies.^15^ These strategies encompass a range of approaches, including but not limited to conventional biologics, fecal microbiota transplantation, genetically engineered microbiome, and next-generation probiotics.^15,16^ While the majority of these biotherapeutic products have only been demonstrated *in vitro*, recent years have seen more concepts advancing to the translational stage.^17^ This progress has contributed to a growing number of clinical trials designed to assess their safety and efficacy in humans.^18,19^ While live bacteria-directed biotherapeutic products are undergoing clinical trials for a diverse array of diseases, such as various solid tumors, chronic kidney disease, hepatic encephalopathy, irritable bowel disease, and ulcerative colitis, only two such products have secured FDA approval.^19^ Ferring’s Rebyota (fecal microbiota, live-jslm) and Seres’ Vowst (fecal microbiota spores, live-brpk) have both been approved for the prevention of recurrent *Clostridioides difficile* infection.^20,21^ These recent surges in clinical trials and venture capitals spending in this field highlights the evident potential of gut bacteria based research on future human health.

A common mechanism by which the gut bacteria manifest these therapeutic activities is through the production of thousands of small molecules and metabolites.^15^ These chemicals function as signaling molecules, accumulating in the gastrointestinal system and reaching distant organs through the blood circulatory system.^22^ Short-chain fatty acids (SCFAs), bile acids, indole derivatives, vitamins, and polyamines stand out as the most dominant known classes of metabolites that regulate the host-microbiota cross-talk.^23^ However, this represents merely the tip of the iceberg as much remains untapped to date. While numerous other live bacteria-directed biotherapeutic products are currently in development, the ongoing research to identify gut bacteria with additional health benefits persists. Thus, the existing knowledge gap underscores the necessity for the current exploration into the therapeutic potential of gut bacteria-derived metabolites.

Many research groups have previously endeavored to study the probiotic properties of human gut bacteria, but the absence of metabolite-oriented studies does not negate their complete bioactivity and therapeutic potential. Thus, the present study aims to isolate and identify some culture-dependent human gut bacteria focusing on their health benefits. Furthermore, a series of *in vitro* evaluations assess the antimicrobial, antioxidant, and anti-thrombotic therapeutic potentials of metabolites derived from these bacteria.

## Materials and methods

**Figure 1.**
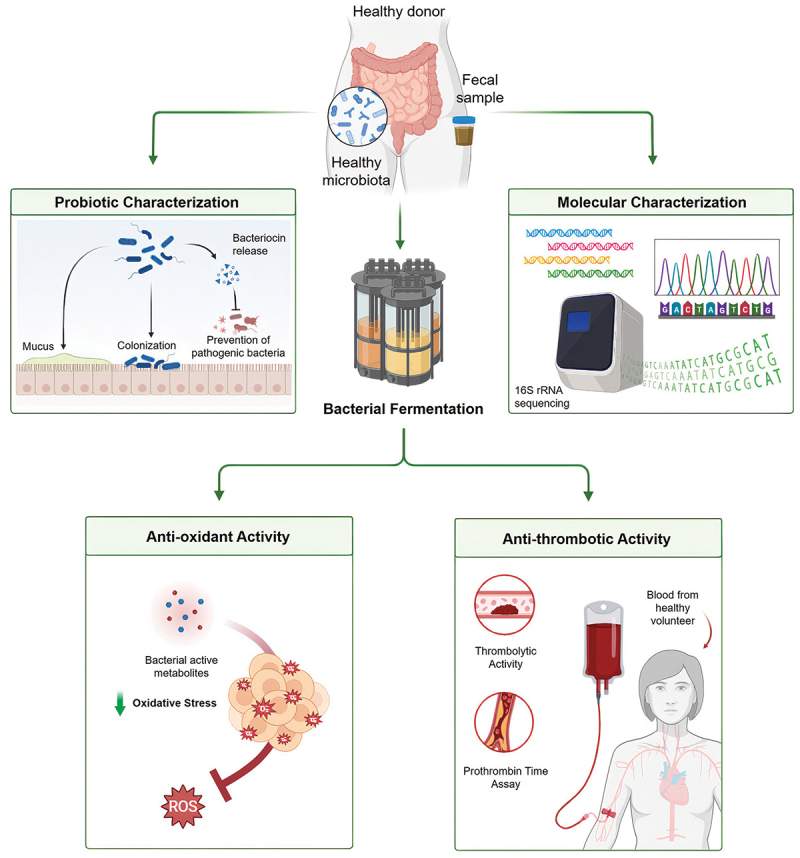
Overview of experimental workflow. This study started by culturing gut bacteria sourcing from fecal samples of healthy volunteers. The bacteria were characterized focusing on their health benefit and identified through 16S rRNA gene sequencing. Simultaneous fermentation of the bacteria yielded metabolite products. These metabolites were further subjected to series of *in vitro* anti-oxidant and anti-thrombotic experiments.

A schematic representation of the study methodology is provided in Figure 1 for clarity and convenience.

### Collecting and processing of fecal samples

Fecal samples were obtained from July 30^th^ to August 7^th^, 2023, from a cohort of forty healthy human volunteers (20 male and 20 female), meeting eligibility criteria outlined in previous report.^24^ Volunteers were aged 18–25, not pregnant, in general good health, refrained from antibacterial medications for the preceding three months, reported no history of tobacco or alcohol use, and had no gastrointestinal or other digestive disorders, as well as no significant medical conditions such as diabetes, heart disease, hypertension, or malignant tumors. Samples were collected using a stool specimen container (JMI Group), sealed with parafilm, stored on ice, and transported to the laboratory.

The collected specimens were prepared following established protocols with minor adjustments.^25,26^ After collection, fecal samples from the forty volunteers were combined into a pooled sample. 1 gram of the pooled sample was suspended in 10 mL of sterile phosphate-buffered saline (PBS, Sigma Aldrich, USA) enriched with 0.1% L-cysteine. The suspension was gently vortexed for 5 min, followed by 10 min of settling at room temperature to allow insoluble particles to settle. The supernatant was further collected for culturing purposes.

### *In vitro* culture

A 100 μL aliquot of the supernatant was inoculated by even distribution onto three different agar media plates. These are Bacterial Growth Medium (BGM),^27^ Brain Heart Infusion (BHI) Medium (Condalab, Madrid, Spain) and MacConkey Agar Medium (Condalab, Madrid, Spain). All agar media were prepared in advance according to manufacturer’s instructions and sterilized. The inoculated plates were incubated at 37°C for 24 to 48 hours, and colony appearance was observed. Following the colony picking strategy outlined by Chang and colleagues,^24^ plates with over 100 colonies were selected. Colonies exhibiting distinct morphologies (variations in color, shape, and size) were subcultured for at least 10 times to establish pure cultures. The pure colonies were transferred to nutrient agar (Condalab, Madrid, Spain) plates and stock cultures were maintained at − 20°C in nutrient broth (Condalab, Madrid, Spain) with 50% (v/v) glycerol. Identifying numbers were assigned based on the specific growth medium.

### Probiotic characterization

#### Acid tolerance

The acid tolerance of isolates was evaluated by assessing viable cell counts.^28,29^ A 10% (v/v) aliquot of each isolate cultures were grown overnight in nutrient broth that were further introduced in freshly prepared nutrient broth adjusted to pH 2, 2.5, and 3 with 1N HCl solution. Samples (100 μL) were collected at 0 h (T_0_) and after a 4-hour incubation (T_1_) at 37°C under 150 rpm agitation. These samples were then plated onto nutrient agar and incubated at 37°C for 24 hours. Acid tolerance was determined by comparing viable bacterial cell counts (expressed as log_10_ CFU/ml) at two different conditions. The relative survival rate was calculated using the formula:^30^

$$\mathrm{Relative}\;\mathrm{Survival}\;\mathrm{Rate}\left(\%\right)=\frac{NT_{1}}{NT_{0}}\times 100$$

Where NT_1_ represents the log_10_ CFU or the number of viable cells after incubation at T_1_, and NT_0_ stands for the log_10_ CFU at T_0_ or the initial number of viable bacteria.

#### Bile tolerance

Bile tolerance of the isolates was determined following established methods with minor adjustments.^30,31^ A 10% (v/v) aliquot of each isolate’s overnight cultures in nutrient broth was prepared and inoculated into freshly prepared nutrient broth, either without bile (control group) or supplemented with 0.1%, 0.2%, and 0.3% (w/v) bile salt (Sigma Aldrich, USA). All groups were incubated at 37°C for 4 hours with gentle shaking at 150 rpm. Subsequently, 100 μL from each sample was spread on nutrient agar plates and incubated overnight at 37°C. After incubation, viable bacterial cell count was expressed as log_10_ CFU/ml, and the relative survival rate was calculated using the formula:

$\mathrm{Bacterial}\;\mathrm{Survival}\;\mathrm{Rate}\left(\%\right)=\frac{N_{c}}{N_{0}}\times 100$

Here, N_c_ represents the log_10_ CFU or the number of viable cells after incubation at different concentrations of bile salt, and N_0_ corresponds to the log_10_ CFU in the control group, representing the initial number of viable bacteria.

#### Antibacterial activity

For initial assessment, cell-free culture supernatant (CFCS) from each isolate was obtained according to previous report^31^ serving as an alternative to bacteria-derived metabolites. The antibacterial activity of the collected CFCS was tested on three pathogenic bacterial strains, consisting of one gram-positive bacterium (*Staphylococcus aureus* ATCC 25,923) and two gram-negative bacteria (*Escherichia coli* ATCC 8739 and *Salmonella typhi* ATCC 13,311), by agar-well diffusion method.^32^

Pure cultures of pathogenic strains were incubated overnight in nutrient broth at 37°C. After incubation, each indicator bacterium was evenly spread on Mueller Hinton Agar (Condalab, Madrid, Spain) plates. The plates were allowed to dry, and wells (5 mm) were created with a sterile borer. Subsequently, separate wells were filled with aliquots of the CFCS from the isolates or distilled water (negative control) and incubated at 37°C for 24 hours. Digital calipers were used to measure the diameter of the zone of inhibition (ZOI), categorized as strong (>20 mm), moderate (11–20 mm), or low (<10 mm) inhibition.^33^

### Fermentation and extraction of active metabolites

A single colony from each isolate was transferred to nutrient broth and incubated overnight at 37°C. Cultures were then inoculated into Fermentation Production Medium (Thermo Fisher Scientific, USA) following the manufacturer’s instructions. Bacterial growth was monitored by measuring the optical density at 600 nm (OD_600_) using a UV/VIS spectrophotometer (Labomed, USA) every hour. Fermentation proceeded in a gyrorotatory shaker (150 rpm) at 30°C until isolates reached their respective stationary phases.

Upon reaching mid-stationary stages, cultures underwent centrifugation (10,000 rpm, 10 min, 4°C) to separate cells. Extracellular bacterial active metabolites (BAM) were filtered through Whatman filter paper no. 1 (Whatman Ltd., England). Ethyl acetate (1:1) was mixed with the filtrates, shaken for 2 hours, and the organic layer was isolated using a separating funnel. The collected organic layer, containing BAM, was incubated at 45°C and dissolved in Dimethylsulfoxide (DMSO, Sigma Aldrich, USA) for subsequent analysis as test samples.

### *In vitro* antioxidant activities

#### DPPH‑free radical scavenging assay

2,2-diphenyl-1-picrylhydrazyl (DPPH) scavenging assay was performed according to standard protocol with minor adjustments.^34^ In a 96-well plate, freshly prepared 0.004% (w/v) DPPH ethanolic solution (pH 5.5) was combined with an equal volume of various concentrations (25–400 μg/mL) of either test samples or standard Quercetin (Loba Chemie, India). After a 1-hour light-free incubation at 25°C, absorbance at 517 nm was measured. DMSO served as a blank instead of test samples or standard. Free radical scavenging activity was calculated as a percentage of inhibition using the equation:^35^

$$\mathrm{Inhibition}\left(\%\right)=\frac{\mathrm{Absorbance}\;\mathrm{of}\;\mathrm{blank}\;-\;\mathrm{Absorbance}\;\mathrm{of}\;\mathrm{sample}}{\mathrm{Absorbance}\;\mathrm{of}\;\mathrm{blank}}\times 100$$

The percentage of inhibition activity was plotted against the concentrations of the samples or standard (μg/mL) to determine the concentration required to scavenge 50% of the DPPH free radical (IC_50_).

#### Reducing power assay

The reducing power of test samples were evaluated in according to standard protocol with minor modification.^36^ Briefly, different concentrations of test samples or standard Quercetin were added with 200 mM PBS (pH 6.6) and 1% potassium ferricyanide in a 96-well plate. Following a 30 min incubation at room temperature, the mixtures were added with 10% trichloroacetic acid and subsequently centrifuged at 3000 rpm for 10 min. The upper layer was mixed with equal volume of 0.1% ferric chloride. The absorbance of this solution was measured at 700 nm and reducing power was determined by the following equation:

$$\mathrm{Reduction}\left(\%\right)=\frac{\mathrm{Absorbance}\;\mathrm{of}\;\mathrm{blank}\;-\;\mathrm{Absorbance}\;\mathrm{of}\;\mathrm{sample}}{\mathrm{Absorbance}\;\mathrm{of}\;\mathrm{blank}}\times 100$$

IC_50_ value was determined by plotting the percentage of reduction activity against the concentration of the samples or standard (μg/mL).

### Anti-thrombotic activity

#### Thrombolytic activity

Thrombolytic activity was assessed following a previously described method.^37^ Freshly collected blood samples were distributed into pre-weighed sterile Eppendorf tubes (500 μL/tube) and incubated for 45 min at 37°C. After clot formation, serum was carefully extracted without disrupting the clot. Each tube with a clot was re-weighed to calculate the clot weight [clot weight (W_3_) = weight of tube with clot (W_2_) – weight of empty tube (W_1_)].

Subsequently, 100 μL of test samples at various concentrations (25–400 μg/mL) were added to tubes containing clots. An equivalent volume of streptokinase (1200 U/mg, Square Pharmaceutical Ltd., Bangladesh) or distilled water served as positive and negative controls, respectively. Tubes were then incubated at 37°C for 90 min and observed for clot lysis. After incubation, the generated fluid was removed, and tubes were re-weighed. The percentage of clot lysis was calculated using the formula:

$$\mathrm{Clot}\;\mathrm{Lysis}\left(\%\right)=\frac{W_{3}-W_{4}}{W_{3}}\times 100$$

Here, W_3_ represents the initial clot weight, and W_4_ signifies the weight of the lysed clot after 90 min incubation.

#### Prothrombin Time (PTT) assay

Prothrombin time was determined as previously reported with minor changes.^38^ Freshly collected blood samples were stored in K2-EDTA tube. The blood samples were subjected to centrifugation at 3000 rpm for 15 min at 4°C, resulting in the isolation of platelet-rich plasma (PRP). These PRP samples were centrifuged again for 10 min to obtain and pool platelet-poor plasma (PPP). Subsequently, 200 µL of PPP was mixed with 100 µL of test samples at various concentrations (25–400 μg/mL) and incubated at 37°C for 30 min. Subsequently, 300 µL CaCl_2_ (25 mM) was added into each reaction tube and the time required to coagulate was recorded with stopwatch. Warfarin (Square Pharmaceutical Ltd., Bangladesh) and distilled water served as positive and negative controls, respectively.

### Molecular characterization

The extraction of bacterial genomic DNA (gDNA) was carried out following established protocols.^39^ The DNA yields and purity were assessed using a UV-Vis NanoDrop spectrophotometer (NanoDrop 2000, ThermoFisher Scientific, Germany) by examining the DNA absorbance ratios at 260 nm/280 nm and 260 nm/230 nm.

16S Barcoding Kit (SQK‐RAB204; Oxford Nanopore Technologies, Oxford, UK) was used according to the manufacturer’s instruction to amplify the 16S rRNA sequence from the bacterial genomes including the following primers:^40^

Forward: *27F (5’-AGA GTT TGA TCM TGG CTC AG-3’)*

Reverse: *1492 R (5’- CGG TTA CCT TGT TAC GAC TT-3’)*

The amplification process was carried out in a thermocycler (Gene Atlas, Astec, Japan) under optimized PCR conditions.^41^ Subsequently, the PCR products were verified on a 1% agarose gel stained with ethidium bromide (Promega Corporation, USA)^42^ and visualized using a manual gel documentation system (InGenius3, Syngene, USA). The amplified gene fragments were purified and sequenced with the same primers from Invent Technologies Ltd., Dhaka, Bangladesh. The raw sequences obtained were analyzed using Chromas software (technelysium.com.au/wp/chromas). Following that, sequence similarity was assessed by comparing them to the NCBI GenBank (https://www.ncbi.nlm.nih.gov/genbank) database using the BLAST algorithm.

### Statistical analyses

All experiments were conducted in triplicates. Data are expressed as mean ± standard deviation (SD). Statistical comparisons of controls and test samples were evaluated by one-way ANOVA by Graphpad prism Version 8 (GraphPad Software, Inc., USA). A *p* value < 0.05 was considered statistically significant.

## Results and discussion

### 16S rRNA sequencing identifies the isolated gut bacteria

**Figure 2.**
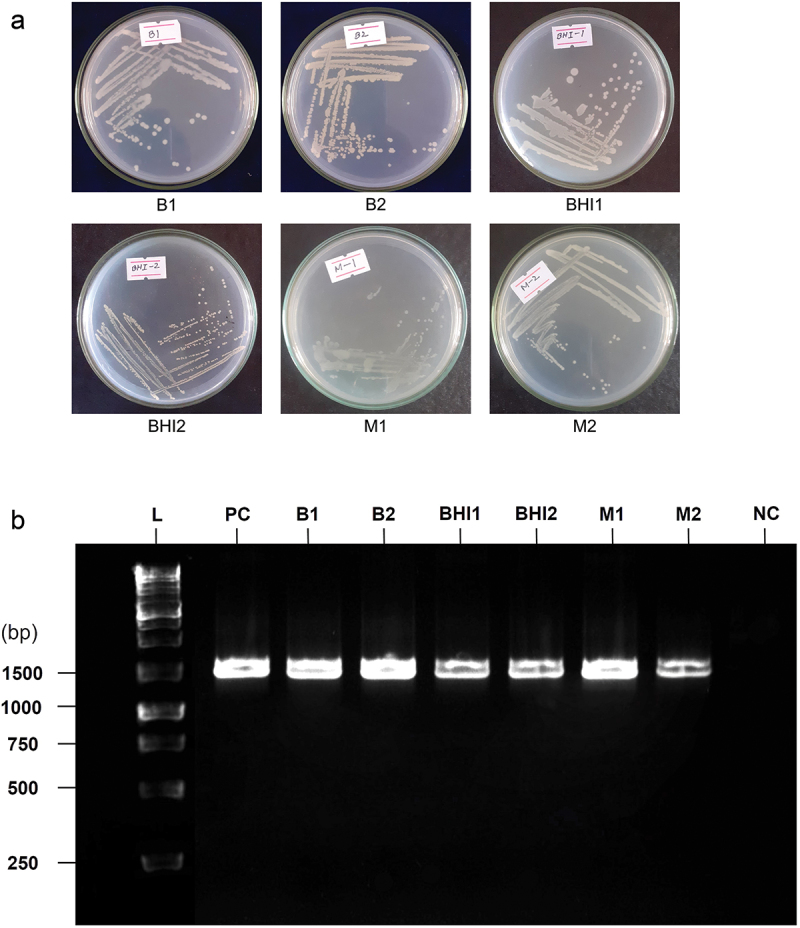
Isolation and identification of bacteria. (a) Growth of pure colonies of the six gut bacteria as follows: B1 and B2 in bacterial growth media, BHI1 and BHI2 in brain heart infusion, M1 and M2 in MacConkey media. (b) PCR amplification with 16S rRNA genes of the six isolates. The bands of high intensity correspond to positive results with positive bands at ~1500 bp of the six bacterial isolates (B1, B2, BHI1, BHI2, M1, M2). L: 1 kb DNA ladder, PC: positive control (16s rRNA fragments of *Escherichia coli* ATCC 8739), NC: negative control (nuclease free water).

The colony picking strategy^24^ resulted in the isolation of six gut bacteria, with two isolates on each specific medium (Figure 2a). Isolates B1 and B2 were cultured on Bacterial Growth Media (BGM). Previous studies have employed this growth medium to culture intestinal bacteria from fecal samples.^43,44,94^ These findings confirmed the abundant growth of *Proteobacteria* and *Firmicutes* in BGM.^43^ Isolates BHI1 and BHI2 were cultured in Brain Heart Infusion (BHI) media, one of the most commonly used media for culturing human gut bacteria.^45,109^ This medium typically supports the growth of *Bacteroidetes*, *Actinobacteria*, or *Proteobacteria*.^43^ While MacConkey media is not a conventional choice for such studies, there have been earlier attempts to culture gut microbiota on this selective medium,^46^ primarily of other species.^47,48^ In this study, M1 and M2 were isolated on this medium.

The extracted gDNA from all six isolates was deemed pure according to the NanoDrop spectrophotometer readings (Table S1). The DNA concentration varied from 89.57 to 127.63 ng/µl. DNA absorbance ratios at 260/280 ranged from 1.74 to 1.99, and at 260/230, the ratios ranged from 2.02 to 2.18. These values ensure the purity of the extracted DNA and validate their suitability for further analyses. Following the amplification of the 16S rRNA genes, the segments were confirmed using agarose gel electrophoresis (Figure 2b). The PCR assay produced DNA amplicons of approximately ~ 1500 base pairs in length, and all the samples exhibited peaks within that region.

The alignment of these 16S rRNA sequences from the isolates against the NCBI GenBank database identified the best-matched bacterial species, as shown in Table 1. Out of the six isolates, two were identified as *Bacillus spp*., two as *Escherichia spp*., and the remaining two as *Lactobacillus rhamnosus* and *Priestia flexa*. While the isolates in our study showed 100% identity to specific *L. rhamnosus*, *B. subtilis*, and *E. coli* strains, confirming their strain specificity, the other three isolates (*P. flexa*, *Bacillus spp*., and *E. fergusonii*) were not a complete match to any known sequence but exhibited high similarity (>96.05%). The partial 16S rRNA sequences of all the isolates are given in Table S2. These isolates are preserved as stock culture at the Microbiology Lab of BGE Discipline at Khulna University, Bangladesh.

**Table 1.** Summary of BLAST result: identified gut bacteria by 16s rRNA gene sequencing.

Bacterial Isolates	Best Matched Organism	Strain	Accession ID	Identity	Query Cover
B1	*Lactobacillus rhamnosus*	IDCC 3201	EF3991.1	100%	100%
B2	*Priestia flexa*	SBAWP2	LC9354.1	96.77%	96%
BHI1	*Bacillus spp.*	KA1	AB8886.1	96.05%	99%
BHI2	*Bacillus subtilis*	HU5	EF1713.1	100%	100%
M1	*Escherichia coli*	ED1a	CU8162.2	100%	100%
M2	*Escherichia fergusonii*	WW10–8	MW2342.1	97%	98.15%

All six identified isolates are commonly recognized components of the gut microbiota. Strains such as *L. rhamnosus* IDCC 3201 or *E. coli* ED1a are well-established probiotics that have undergone trials for treating various disease conditions.^49,50^ *L. rhamnosus* IDCC 3201, recognized as a GRAS bacterium, was previously isolated from breast-fed infant feces.^49,51^ Other strains of *L. rhamnosus* have also been routinely isolated from adult fecal samples.^52^ As lactic acid bacteria, these strains exhibit a wide range of immunoregulatory activities when used as a food supplement.^53^ In a previous study, Jeong and colleagues concluded that the oral administration of tyndallized *L. rhamnosus* IDCC 3201 showed therapeutic effects in children with atopic dermatitis.^49^ *E. coli* ED1a is another nonpathogenic and human-specific gut colonizer that is well adapted to the healthy human gut.^54,55^ This particular strain has been previously isolated in feces of healthy young individuals.^54,56^ However, existing research on *E. coli* ED1a primarily focuses on animal models.^50,57^ *P. flexa*, belonging to *Firmicutes*, is a gram-positive bacterium, previously known as *Bacillus flexa*.^58^ Although there is limited information available about the *P. flexa* SBAWP2 strain, other strains of this species have been discovered in soil and coastal environments.^59–61^ However, the recent findings of Deswal and colleagues support our identification of *P. flexa* as a new bacterial strain from healthy human feces.^62^ Consequently, our findings contribute to the understanding that *P. flexa* may be regarded as gut commensals.^62^ The spore-forming genus *Bacillus* is ubiquitous in nature, commonly isolated from soil or water, but it has also been identified in the gut of insects and mammals.^63^ Recent studies have characterized spore-forming *Bacilli* isolated from the human gut.^64,65^ Various strains of *B. subtilis*, including strain HU5, have been identified and considered as a part gut microbiota.^64^ Existing research on *B. subtilis* primarily focuses on its effects in improving digestive and intestinal health.^66,67^ *Escherichia fergusonii* is characterized as a rare opportunistic pathogen in humans.^68^ However, earlier studies have reported the nonpathogenic strains of *E. fergusonii* isolated from animals and waste-water sources.^69–71^ Compared to its isolation from individuals with infections, finding *E. fergusonii* in healthy human is not frequent.^72^ Previous reports have not firmly established its regular presence in the gut microbiome of healthy individuals but it’s not completely uncommon.^73,74^ In accordance with that, our study confirms the occurrence of *E. fergusonii* in healthy humans, though further investigation is needed to fully understand its prevalence in this population. Thus, the findings of this study are consistent with earlier reports and also provide a novel avenue for understanding *P. flexa* and *E. fergusonii*.

### Identified gut bacteria exhibit probiotic-like characteristics

**Figure 3.**
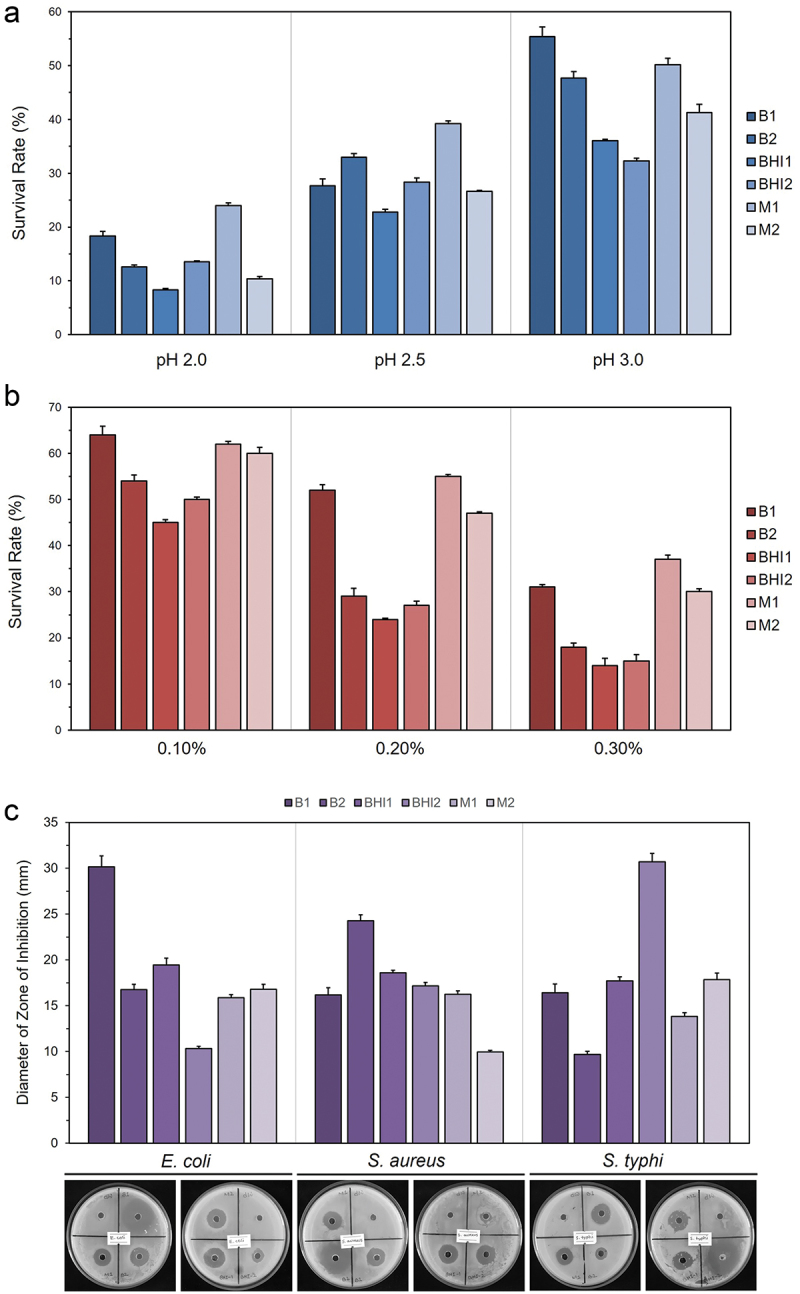
Probiotic characteristics of the six isolates. (a) Bacterial tolerance to acidic conditions. The bar graph illustrates the survival rate (%) of each isolate under varying pH levels. (b) Bar graph represents the bacterial resistance to bile salt concentration. Survival rates (%) of the isolates are plotted against three different concentration of bile salt. (c) Bactericidal effect of the six isolates against three pathogenic bacteria. The figure visually represents the zones of inhibition in petri dishes, accompanied by a bar graph indicating the diameter of the zones of inhibition (in mm) produced by each isolate. Experiments were performed in triplicate. The results are presented as mean ± SD. B1: *Lactobacillus rhamnosus*, B2: *Priestia flexa*, BHI1: *Bacillus spp*., BHI2: *Bacillus subtilis*, M1: *Escherichia coli*, M2: *Escherichia fergusonii.*

In this study, we considered tolerance to acidic conditions, resistance to bile salts, and *in vitro* antimicrobial activities as probiotic characteristics. Although the survival rate of the six isolates were positively correlated with the pH values, the trend was different in each condition (Figure 3a). The differences in survival rates among the isolates under varying pH conditions might be attributed to strain-specific variations in acid tolerance mechanisms. Some bacteria could possess a more efficient proton pump system or enhanced production of acid-neutralizing compounds at intermediate pH levels.^75^ Additionally, specific genetic factors may play a role in these variations.^76^ The fluctuation observed at pH 2.5 may indicate an optimal threshold for certain strains to activate their acid tolerance responses.

Isolates B1 and M1, identified as *L. rhamnosus* and *E. coli*, respectively, and most similar to established probiotic strains, exhibited the highest survival rate under the lowest pH concentration. These findings are consistent with earlier reports.^28,77,78^ Isolate B2, identified as *P. flexa*, also exhibited a higher survival rate at pH 2.0 and pH 2.5. Although this is the first study to explore the acid tolerance of *P. flexa*, a previous study by Nithya and colleagues reported that a related strain, *B. flexus* Hk1 had a much higher acidic survival rate than that of *P. flexa*.^79^

All six isolates demonstrated the ability to survive in the presence of varying concentrations of bile salts (Figure 3b). Consistent with previous findings, *L. rhamnosus* exhibited the highest survival rate (64 ± 1.83%) in 0.10% bile salts after 4 hours of incubation.^28,80^ However, isolates M1 and M2, identified as *E. coli* and *E. fergusonii*, respectively, outperformed the survival rate of *L. rhamnosus* at higher concentration of bile salt. This result could be attributed to the presence of an additional outer membrane in gram-negative bacteria, providing further protection against harsh conditions. At 0.30% of bile salt, isolates BHI1 and BHI2, belonging to *Bacillus spp*., were found to be weakly tolerant. The relationship between *Bacillus spp*. and bile salt is complex.^81^ While some *Bacillus* species can indeed struggle with bile, others demonstrate remarkable tolerance.^81,82^ These results align with observations in other cultures of *B. subtilis* and *B. megaterium*.^79,83,84,^

The bactericidal effect of the six isolates against three pathogenic bacteria is presented in Figure 3c. The findings revealed a broad spectrum of antagonistic activity among the gut bacteria. The CFCS of isolated *B. subtilis* exhibited the highest ZOI value against *S. typhi* ATCC 13,311, followed by the CFCS of *L. rhamnosus* against *E. coli* ATCC 8739. These findings are in agreement with Chae and colleagues, who documented the anti-pathogenic activities of *L. rhamnosus* IDCC 3201 CFCS against *S. aureus* ATCC 25,923 and *S. Typhi* ATCC 13,311.^51^ Recent studies have also confirmed that *Lactobacillus spp*. can exhibit these anti-pathogenic activities through the production of antimicrobial agents, primarily bacteriocins, competition for nutrients, or enhancement of host immune response.^85,86^ Meanwhile, spore-forming probiotic *Bacillus* have received extensive interests for their activity against enteric bacterial infections.^87^ The products or metabolites derived from various strains of *B. subtilis* have been studied for decades to identify antimicrobial components against multi-drug resistant bacteria or pathogenic fungi.^88,89^ Ramasubburayan and colleagues previously reported the antibacterial activity of metabolites derived from mangrove associated *B. subtilis* against *S. aureus* and *S. Typhi.*^90^ However, the gut associated *B. subtilis* of this study exceeded the antimicrobial potential of mangrove-associated *B. subtilis* in terms of ZOI. Although the exact mechanisms behind antimicrobial *Bacillus* remain unexplored, many reports confirm its mode of action by producing large number of antimicrobial metabolites, peptides, enzymes, antibiotics, or bacteriocins targeting the cell wall, cell membrane, or intracellular processes.^88,91^

The CFCS of *P. flexa* was another isolate that exhibited strong inhibition zone against *S. aureus* ATCC 25,923. This finding is consistent with previous reports, although earlier studies indicated slightly lower inhibition of *S. aureus*.^92,93^ This difference could be attributed to the direct integration of two-week-old cultured bacteria into pathogenic organisms, resulting in less efficiency than the current approach.^92^ Interestingly, *E. fergusonii* being a rare opportunistic bacterium itself, it could moderately inhibit the growth of *E. coli* ATCC 8739 and *S. typhi* ATCC 13,311, and also inhibited *S. aureus* ATCC 25,923 to a lesser extent. To the best of the authors’ knowledge, this is the first report to claim the bactericidal effect of *E. fergusonii*. Thus, the finding prompts the notion that *E. fergusonii* may contribute to gut health by competing with other pathogenic bacteria. This study also revealed that the isolated commensal *E. coli* was capable to outgrowth the pathogenic strain of the same species. This result is consistent with the report of Fang and colleagues, who suggested this mechanism to be driven by the secretion of extracellular protease and chaperone.^94^ Thus, these findings strongly recommend further isolation and characterization of the metabolites derived from these gut bacterial isolates. These metabolites may contain promising antibacterial compounds against pathogenic strains and can be further scale-up for understanding their in-depth molecular mechanisms. Simultaneously, the survival rates of these bacteria under harsh condition indicate their potential as live biotherpaeutics.

### Metabolites derived from gut bacteria exhibit anti-oxidant potential

**Figure 4.**
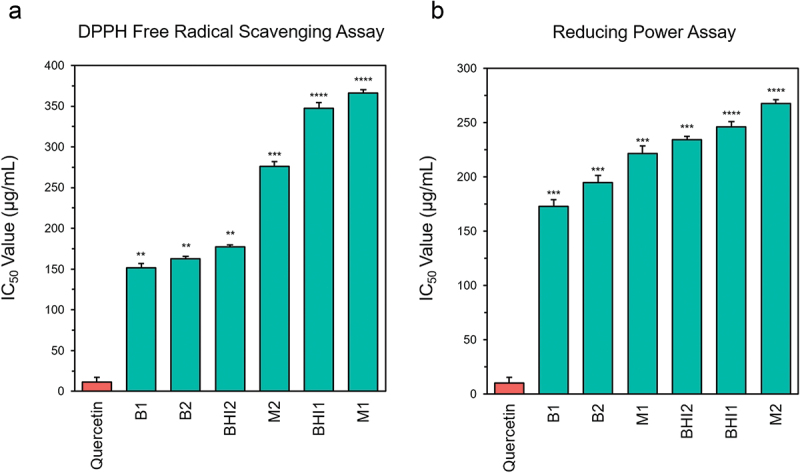
Anti-oxidant potential of the metabolites derived from the six gut bacterial isolates. (a) Bar graph represents the half-maximal inhibitory concentration (IC_50_) in μg/mL of Quercetin and six sets of bacteria-derived metabolites on the free DPPH radical scavenging activity. (b) IC_50_ values of Quercetin and the bacteria derived metabolites are expressed as bar graphs. Experiments were performed in triplicate and data are shown as mean ± SD. Level of significance was determined by one-way ANOVA. **p < 0.01, ***p < 0.001, ****p < 0.0001. B1: *Lactobacillus rhamnosus*, B2: *Priestia flexa*, BHI1: *Bacillus spp*., BHI2: *Bacillus subtilis*, M1: *Escherichia coli*, M2: *Escherichia fergusonii*.

Free radical scavenging-based experiments utilize stable radicals such as DPPH or ferric ions as oxidant molecules, predicting the ability of treatments to inhibit active oxidation. The results and subsequent calculation of IC_50_ values suggest that the metabolites derived from the bacterial isolates are strong antioxidants (Table S3, Table S4). The metabolite sets demonstrated varying IC_50_ values, ranging from 151.47 ± 5.43 μg/mL to 365.95 ± 4.16 μg/mL in the DPPH-based assay and from 172.65 ± 6.41 μg/mL to 267.53 ± 3.54 μg/mL in the reducing power assay. Metabolites from *L. rhamnosus* and *P. flexa* exhibited the lowest IC_50_ values in both assays (Figure 4). *E. coli* and *E. fergusonii* metabolites exhibited a slightly dissimilar trend in the two assays. While *E. coli metabolites* had the highest IC_50_ value in the reducing power assay, it exhibited moderate antioxidant potential in the DPPH-based assay. *E. fergusonii* metabolites exhibited the highest IC_50_ value in the DPPH-based assay but moderate in reducing power assay. Other *Bacillus spp*. isolates demonstrated moderate activity in both assays.

Previous studies on the antioxidant activity of gut bacterial metabolites have been limited, but our findings are in agreement with the claims of Wu and colleagues, who proposed that the gut microbiota is a robust antioxidant system.^95^ According to Uchiyama and colleague, gut bacteria can increase reactive sulfur species levels and reinforce host antioxidant capacity.^96^ Earlier reports confirm lactic acid bacteria exerting strong antioxidant activity.^97,98^ *L. rhamnosus* from diverse sources has been studied *in vivo* under physical and oxidative stress, demonstrating its ability to increase antioxidant levels, enhance survival of human mesenchymal stem cells, reduce anxiety levels and neutralize the effects of reactive oxygen species.^99–101^ While intracellular cellular extracts and exopolysaccharides of *L. rhamnosus* were previously evaluated for their antioxidant activity,^102,103^ this is the first study to report the antioxidant level of metabolites from gut *L. rhamnosus*. However, several antioxidant metabolites have been previously characterized from many *Lactobacillus* species. Ahire and colleagues reported folate-producing *L. helveticus*.^104^ Folate can accept one-carbon units from donor molecules. Glutathione, a non-enzymatic antioxidant, has been reported in *L. fermentum*.^105^ Other species have been reported to produce butyrate that can suppress hepatic oxidative stress.^106^ In the study by Mohammad and colleagues, oral administration of *L. acidophilus* enhanced vitamin B12 in children, indicating an improved oxidative status.^107^

Although there is no existing report on the antioxidant potential of *P. flexa*, various *Bacillus* species have been recognized as elite sources of antioxidants. The study of Duc and colleagues reported varying antioxidant activities of different *B. subtilis* strains^108^ but our results are mostly in agreement with the findings of Ramasubburayan and colleagues.^90^ Large number of bioactive secondary metabolites, pigments, and peptides derived from *Bacillus* strains have yielded this robust antioxidant activities.^109–111^ Such activities lead to reduced intestinal inflammation and positively modulate the gut microbiota.^112^ In a previous study, Sy and colleagues identified carotenoid-producing *Bacillus spp*., with carotenoids being efficient lipophilic antioxidants capable of inhibiting iron-induced lipid peroxidation.^113,114^ Similarly, alpha-glucosidase inhibitors or surfactins derived from *B. subtilis* have been shown to enhance antioxidant activities and protect human normal keratinocytes.^110,115,116^ *E. coli* can activate various systems to protect cells from oxidative stress, primarily by up-regulating multiple genes in response to free radicals.^117^ Extracellular products of *E. coli* also exhibit strong antioxidant activity.^118^ Moreover, *E. coli* has been reported to produce diverse bioactive metabolites, including lycopene.^119^ This tetraterpene compound is a potent antioxidant and has demonstrated efficacy in addressing various disease conditions.^120^

Therefore, the findings of this study offer preliminary insights into the antioxidant activities of the gut bacteria-derived metabolites. Further characterization of these metabolites may reveal the underlying molecular mechanisms addressing oxidative stress. These compounds could potentially serve as solutions to enhance the oxidative stability of human cells and prevent oxidative instability and subsequent inflammatory diseases.

### Gut bacteria derived metabolites can lyse blood clots and prolong blood coagulation time

The existing body of empirical evidence has firmly established the role of gut bacteria extending beyond the confines of the human GIT. Numerous studies have documented their influence on diverse physiological systems, including the modulation of blood-brain barrier integrity, promotion of brain health, and even implications in hypertension.^121,122^ Recent findings have further asserted that proteins produced by gut bacteria may contribute to reducing the risk of heart diseases.^123^ While the impact of gut bacteria (or their products) is certain on blood, this study reports the effect of metabolites derived from the identified gut bacteria on blood coagulation and clotting.

**Figure 5.**
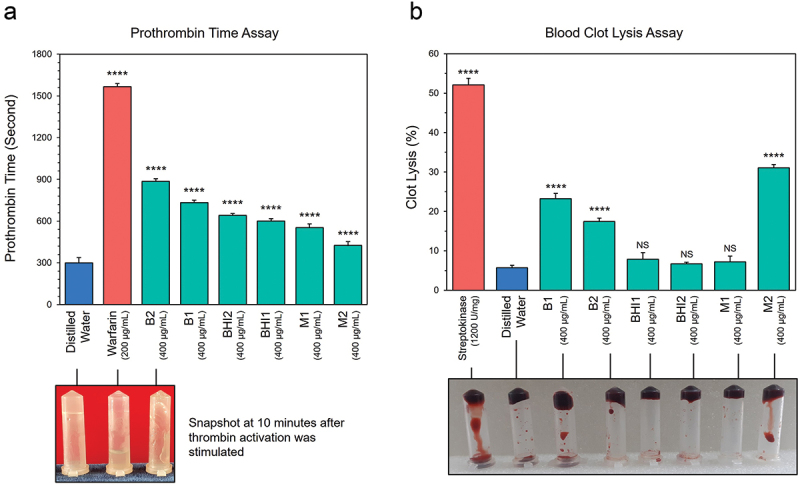
Anti-thrmobotic potential of the metabolites derived from the six gut bacterial isolates. (a) Bar graph represents the time required to form prothrombin induced fibrin clots by 400μg/mL of test samples, 200μg/mL of warfarin and distilled water. Snapshot after 10 min of experiment starting point shows that plasma treated with test sample (B2: *P. flexa*) remained semi-fluidic upon inversion, while warfarin treated plasma remained liquid and distilled water treated plasma remained as non-fluidic. (b) Bar graph represents the % of blood clot lysis by 400μg/mL of test samples, 1200 U/mg of streptokinase and distilled water. Visual representation of the clot-lysis of blood samples where tubes were inverted after dissolution of the clots. Experiments were performed in triplicate and data are shown as mean ± SD. Level of significance was determined by one-way ANOVA. ****p < 0.0001. B1: *Lactobacillus rhamnosus*, B2: *Priestia flexa*, BHI1: *Bacillus spp*., BHI2: *Bacillus subtilis*, M1: *Escherichia coli*, M2: *Escherichia fergusonii*.

The PTT assay results demonstrate the anti-thrombotic potentials of the crude metabolites derived from the six isolates, showing a highly significant prolongation of coagulation time by inhibiting calcium-induced thrombin activation (Table S5). Notably, metabolites from *P. flexa* (886 ± 19 sec) and *L. rhamnosus* (733 ± 17 sec) were among the best performing, competing with the positive control warfarin (Figure 5a). However, it is worth mentioning that a concentration of metabolites twice as high as that of the control was needed to achieve this competency.

The diverse metabolites produced by gut microbiota significantly influence platelet activity, endothelial function, and inflammation.^125^ Among these, short-chain fatty acids (SCFAs) such as acetate, propionate, and butyrate have been reported to modulate platelet function and improve vascular health.^124^ Studies indicate that SCFAs regulate the release of platelet-activating factors and inhibit platelet aggregation.^126^ Additionally, gut bacteria produce secondary bile acids, including deoxycholic acid and lithocholic acid, which exert anti-inflammatory effects.^127^ These actions may indirectly lower thrombosis risk by mitigating endothelial dysfunction and inflammation-driven platelet activation. Furthermore, indole derivatives like indole-3-propionic acid exhibit antioxidant and anti-inflammatory properties that support vascular health and thrombosis prevention.^128^ Multiple strains to *L. rhamnosus* and their supernatant exhibited protective effect against myocardial dysfunction in murine model.^129,130^ While earlier *in vivo* findings revealed *L. rhamnosus* peptidoglycan modulating the procoagulant state, this study is the first to report the anti-thrombotic potential of *L. rhamnosus* metabolites.^131^ Similarly, the lack of scientific research directly investigating the thrombolytic activity of *P. flexa* offers us the fresh opportunity to report the anti-thrombotic activity of *P. flexa* metabolites. On the other hand, a set of highly significant serine protease inhibitors of thrombotic processes, and thrombolytic enzymes have been previously characterized from *B. subtilis* .^132–134^ Findings from Ghoneim and colleagues confirm the positive effect of exopolysaccharides from *B. subtilus* on atherogenic indices.^135^ In consistent with those findings, our results confirm the thrombolytic effect of *B. subtilis* metabolites. Scientific literature also documents the enzymatic thrombolytic potential of *Escherichia spp*. Serine proteases, such as ecotin or EspP, have been routinely found to be produced by *E. coli*.^136,137^ These findings are consistent with our study but at the metabolite level.

The blood clot lysis assay demonstrated a dose-dependent effect, higher concentration of bacteria-derived metabolites lysed more content of clots (Table S6). Metabolites from *Bacillus spp*. and *E. coli* had no significant effect compared to distilled water, whereas metabolites from *L. rhamnosus* and *P. flexa* demonstrated highly significant clot-lysing ability (Figure 5b). Remarkably, metabolites derived from *E. fergusonii* exhibited the highest clot lysis potential (31.08 ± 0.85%), closely rivaling that of streptokinase (52.08 ± 1.7%). However, previous case studies had associated *E. fergusonii* with hemolytic uremic syndrome, characterized by thrombosis.^138,139^ This bacteria also actively contributes to Trimethylamine-N-oxide production, which is a major pro-thrombotic molecule.^140^ Despite this established findings, our *in vitro* results provide novel insights, suggesting that *E. fergusonii* metabolites may also possess antithrombotic potential. While this study is limited in exploring the underlying molecular mechanism, strain-specific effects could be a possible explanation for this attribute.

Consequently, this study underscores the importance of further characterizing the metabolites derived from *L. rhamnosus*, *P. flexa*, and *E. fergusonii*. These metabolites might hold strong potential against atherosclerotic cardiovascular diseases or coronary disorders.

## Limitations and future recommendation

There are some notable limitations in the scope of the current study that should be addressed. The present study takes into account of some culture-dependent aerobic gut bacteria that were chosen by the colony picking strategy. Therefore, the bacterial entities presented here should not be considered a representation of the entire human gut microbiome, rather we provided a general therapeutic potential of some human gut bacteria that can be readily cultured and scaled under aerobic conditions.

Additionally, the diversity of the microbiome can vary significantly with geography and diet. The volunteers for this study were from a specific geographical region and maintained a healthy lifestyle, which allowed us to capture a general snapshot of the gut bacteria and its therapeutic potentials. However, this study does not encompass the complete gut microbiota composition.

Another notable limitation is the lack of detailed characterization of the metabolites derived from gut bacteria responsible for the observed effects. While our findings confirm promising therapeutic potential, the chemical structures and mechanisms underlying these effects remain undetermined. The next stage of this research will involve a comprehensive metabolomics approach to precisely identify and quantify the metabolites responsible for the observed bioactivities. This will utilize advanced techniques, such as omics data analysis, LC-MS or NMR spectroscopy, to characterize the metabolites in greater detail.

On that note, we considered individual bacteria in a controlled setting rather than within the complex microbial communities found in living systems, where environment-microbe, host-microbe, and microbe-microbe interactions play significant roles. While our study demonstrates promising bioactivity *in vitro*, extending to *in vivo* or animal models remains a major challenge to be addressed in future research.

Finally, safety concerns regarding these six isolates must be acknowledged for therapeutic applications. While these strains demonstrated beneficial effects *in vitro*, their safety for therapeutic use in humans requires rigorous evaluation. This study does not advocate using these isolates directly as live biotherapeutics but suggests comprehensive analyses to assess virulence, identify resistance genes, and consider the modification or improvement of strains where necessary. As an alternative to live biotherapeutics, we may consider isolating and administering the purified metabolites to mitigate the risk of pathogenicity. Further strain-specific investigations to better understand the interplay between species-wide traits and individual strain behavior in mediating therapeutic effects is necessary. It is also essential to compare these findings with commercially available and established probiotics and biotherapeutics. Nevertheless, this study provides a foundational insight into the potential roles of gut bacteria and their metabolites in host-microbiome crosstalk from a therapeutic perspective. We recommend further research toward developing novel biotherapeutics based on these gut bacteria.

## Conclusion

**Figure 6.**
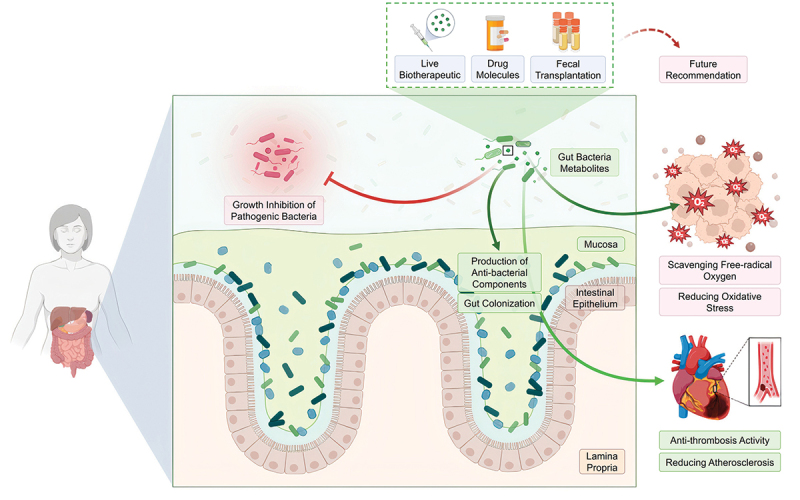
A model illustration reflecting the overall findings of this study, depicting how the isolated gut bacteria might exert therapeutic potentials. Primarily, the gut-habituated bacteria may inhibit the presence of other pathogenic bacteria. This inhibition can occur through various mechanisms, such as competition for nutrients, colonization of gut cells, or secretion of bacteriocin or other antimicrobial agents. Metabolites derived from the isolated bacteria exhibited potential to scavenge free radicals. Hence, these metabolites could be antioxidants, reducing oxidative stress of our cells. These metabolites also lysed blood clotting and prolonged blood coagulation time *in vitro*, suggesting a role beyond gut. Perhaps these mechanisms protect our body from atherosclerosis or coronary disorders. Probably these metabolites could serve as next generation therapeutics. Maybe these metabolites are the language through which the gut bacteria communicate with our cells.

In summary, this preliminary study shed light on some novel therapeutic aspects of human gut bacteria and their derived metabolites. These aerobically culture-dependent bacteria exhibited probiotic-like properties and their metabolites exerted significant benefits during *in vitro* experimentation of antibacterial, antioxidant, and anti-thrombotic qualities (Figure 6). These are essential mechanisms in preventing microbial infections, improving oxidative stability, reducing inflammatory diseases, atherosclerosis and thrombosis. Taken together, these findings contribute to our understanding of the role of the isolated gut bacteria and their derived metabolites. The next stage of this study will call for a metabolomics approach to identify the potential metabolites as putative contributors to the therapeutic qualities. These will provide critical mechanistic insights of the metabolites and their inter-molecular interactions. From a therapeutic perspective, further optimization of production, stabilization, and *in vivo* investigation should be carried out to advance the development of gut bacteria-based novel biotherapeutics.

## Supplementary Material

Supplementary Tables.xlsx

## Data Availability

The authors confirm that the data supporting the findings of this study are available within the article [and/or] its supplementary materials.
